# Conclusion or Illusion: Quantifying Uncertainty in Inverse Analyses From Marker-Based Motion Capture due to Errors in Marker Registration and Model Scaling

**DOI:** 10.3389/fbioe.2022.874725

**Published:** 2022-05-25

**Authors:** Thomas K. Uchida, Ajay Seth

**Affiliations:** ^1^ Department of Mechanical Engineering, University of Ottawa, Ottawa, ON, Canada; ^2^ Department of BioMechanical Engineering, Delft University of Technology, Delft, Netherlands

**Keywords:** inverse dynamics, inverse kinematics, joint power, marker placement, marker registration, modeling uncertainty, OpenSim, scaling

## Abstract

Estimating kinematics from optical motion capture with skin-mounted markers, referred to as an inverse kinematic (IK) calculation, is the most common experimental technique in human motion analysis. Kinematics are often used to diagnose movement disorders and plan treatment strategies. In many such applications, small differences in joint angles can be clinically significant. Kinematics are also used to estimate joint powers, muscle forces, and other quantities of interest that cannot typically be measured directly. Thus, the accuracy and reproducibility of IK calculations are critical. In this work, we isolate and quantify the uncertainty in joint angles, moments, and powers due to two sources of error during IK analyses: errors in the placement of markers on the model (marker registration) and errors in the dimensions of the model’s body segments (model scaling). We demonstrate that IK solutions are best presented as a distribution of equally probable trajectories when these sources of modeling uncertainty are considered. Notably, a substantial amount of uncertainty exists in the computed kinematics and kinetics even if low marker tracking errors are achieved. For example, considering only 2 cm of marker registration uncertainty, peak ankle plantarflexion angle varied by 15.9°, peak ankle plantarflexion moment varied by 26.6 N⋅m, and peak ankle power at push off varied by 75.9 W during healthy gait. This uncertainty can directly impact the classification of patient movements and the evaluation of training or device effectiveness, such as calculations of push-off power. We provide scripts in OpenSim so that others can reproduce our results and quantify the effect of modeling uncertainty in their own studies.

## 1 Introduction

In human movement science and biomechanics, optical motion capture (mocap) is the most common strategy for collecting high-precision movement data. Small markers (either passive photo-reflective or active infrared-emitting) are placed on the skin or tight-fitting clothing of a test subject. The three-dimensional trajectories of the markers are measured (“captured”) by a calibrated set of high-resolution cameras as the subject moves through space (specifically, the calibrated “capture volume”) and over time. Markers are placed on bony landmarks to identify anatomical axes and to minimize the effect of skin movement and other soft-tissue artifacts. Within a well-calibrated capture volume, one can use stereophotogrammetry (Cappozzo et al., 2005) of multiple camera views to measure the position of a marker to within a fraction of a millimeter (Uchida and Delp, 2020). The resulting spatial marker trajectories are then used to estimate the motion of the underlying bones; with an appropriate link-segment model and measured external forces (e.g., from strain gauges or force plates), joint kinetics and energetics can be computed.

Joint angles are estimated from mocap data by defining anatomical reference frames and computing the relative orientation between frames that are fixed to adjacent body segments. In clinical applications, we often distinguish between typical and atypical movements based on the differences in the trajectories of a patient’s joint angles compared to those of healthy or typically developing controls. For example, the minimum knee flexion angle during stance can be used to distinguish between typically developing walking gait (below 15°) and mild (15–30°), moderate (30–50°), and severe (above 50°) crouch gait in children with cerebral palsy (Steele et al., 2013). Therefore, uncertainty in estimating joint angles could result in different classifications and treatment strategies.

The net joint moments can be estimated from joint angles in combination with a link-segment model, given appropriate model dimensions, body segment inertias, and applied external loads. Joint angles are typically processed (e.g., filtered and interpolated) to estimate joint velocities and accelerations *via* numerical differentiation. When combined with measurements of external forces, the joint angles, velocities, and accelerations enable us to estimate the net joint moments *via* an inverse dynamic analysis from the equations of motion of the link-segment model (Crowninshield et al., 1978; Kuo, 1998). From estimates of joint moments and velocities, we can then estimate joint powers and can quantify both external and internal work *via* numerical integration. Collectively, mocap can provide a rich set of kinematic and kinetic data with which we can understand human and animal movement; however, the inherent uncertainties in the calculated joint angles and derived quantities are rarely quantified or even acknowledged.

Joint angles calculated from mocap data and joint moments calculated from an inverse dynamic analysis are frequently treated and referred to as experimental measurements (e.g., (Favre et al., 2008; Fiedler et al., 2014; Schmitz et al., 2015)), and the mean and standard deviation of each of these “measures” are often considered across trials and individuals in the statistical analysis of a study. Unfortunately, these are not measured quantities but, rather, computed quantities whose reliability depends on the underlying models and processing techniques (Kainz et al., 2017a). While the spatial position of a marker may be known precisely (e.g., to within a fraction of a millimeter) and our kinematic and inverse dynamic equations are mathematically exact, there remains a major source of error and uncertainty in all mocap marker–based studies: the model must be correctly calibrated. In this paper, we focus on two aspects of calibration: the locations of the markers on each body segment of the model, known as “marker registration” (Dunne et al., 2021), and the dimensions of the model’s body segments, known as “marker-based model scaling” (Delp et al., 2007; Kainz et al., 2017b).

Constrained inverse kinematics (IK) methods, and particularly those that apply a least-squares fit using optimization, highlight the need to study the effects of model scaling (Reinbolt et al., 2005; Koller et al., 2021) and marker registration (Dunne et al., 2021). When markers are carefully placed on a model to match the placement of physical markers on a participant’s body, the model’s dimensions are correctly adjusted, and the model’s joint axes are rotated to represent bony deformities (e.g., (Arnold et al., 2001; Hicks et al., 2007; Veerkamp et al., 2021)), then the distances between model markers and their experimental counterparts can be minimized (Lu and O’Connor, 1999). IK yields a single set of joint angles that minimizes the difference (error) between model and experimental markers at each instant in time. For most models and studies of human movement, the calculated joint angles are considered to be reliable if the root-mean-squared marker error is within 1 cm across the entire movement (Hicks et al., 2015).

When performing experiments, one may overlook errors due to marker registration if a model or optimization method is not (explicitly) used to compute joint angles. Accordingly, one may infer that their analysis methods are immune to uncertainties in marker registration. Unfortunately, this is generally false (Schwartz et al., 2004). When markers are used to define a reference frame, an underlying model is already assumed, which treats each body segment as free-floating with respect to its neighbor (unconstrained inverse kinematics; (Dunne et al., 2021)). Although the orientation of a reference frame defined by three or more markers may be precise and accurate, the orientation of the underlying bone still depends on how the markers were registered with respect to the bone. In this unconstrained case, shifting marker positions relative to the bone does not yield marker errors but the estimated joint angles are directly affected (Osis et al., 2015). For example, transverse-plane angles computed at the knee and ankle during running have been found to vary by 7.59° for every 1 cm of marker registration error (Osis et al., 2016). When an underlying model is composed of unconstrained segments, it represents a maximal set of degrees of freedom that can mask errors by way of overfitting, since the model can achieve perfect correspondence with the experimental markers when there are three or fewer markers per segment. To illustrate this point, imagine incorrectly labeling markers that are used to define an anatomical reference frame (Uchida and Delp, 2020). There would be no marker errors but the computed angles would undoubtedly be incorrect due to the misdirected joint axis of the incorrect anatomical frame. While this is an extreme case, where the error would be large and therefore noticeable, similar but smaller (and less obvious) errors are introduced due to imprecise and inconsistent placement of experimental markers on bony anatomical landmarks on a participant’s body. It is a reality that placing markers on a subject is not a precise task (Osis et al., 2016); despite one’s best efforts to place markers consistently across individuals and in agreement with standardized methods used by other experimentalists (Cereatti et al., 2017), the natural anatomical (bone shape) and morphological (body composition) differences between individuals makes it extremely difficult to identify anatomical landmarks consistently to within 1 cm. Indeed, from session to session, placement of markers in the identical location with respect to the bone is not possible, which results in different joint angle calculations even if nothing about the subject’s motion has changed (Della Croce et al., 2005). This is particularly important to note in studies that aim to detect relatively small changes in kinematics over time.

Alternatively, a model can be defined to include only the degrees of freedom that are physically permissible by the skeletal structure of the joints. For example, there is very limited actual knee varus–valgus motion in human walking (less than 2° (Giphart et al., 2012)) and “measured” varus–valgus is, in fact, mostly cross-talk with knee flexion due to an approximate knee flexion–extension axis defined by markers (Woltring, 1994; Della Croce et al., 2005; Jensen et al., 2016). Consequently, a model without the knee varus–valgus degree of freedom will incur marker errors during gait that will include the inability to capture some real movement (±2°); however, these errors are much less than typical cross-talk errors, which are consistently on the order of 8–15° (Jensen et al., 2016).

Regardless of the underlying model (including unconstrained models with free-floating segments), marker registration has a direct effect on the computed joint angles. Furthermore, the dimensions of the model (the distances between anatomical reference frames) also affect estimates of kinetics (Reinbolt et al., 2007; Riemer et al., 2008; Pàmies-Vilà et al., 2012; Koller et al., 2021) and energetics (e.g., joint powers). While previous studies have investigated the effect of model uncertainty on kinematics and kinetics (Reinbolt et al., 2007; Valente et al., 2014; Myers et al., 2015), in this study we aim to quantify the effect of marker registration and model scaling errors on joint angles, moments, and powers. We ignore the errors introduced by soft-tissue artifacts (Stagni et al., 2005) in order to isolate the effect of marker registration and scaling alone, since soft-tissue motion will increase uncertainty in quantities of interest that are computed from marker-based mocap data when typical analysis strategies are used. In addition, the amount of error due to soft-tissue motion will differ between individuals and parts of the body, and it is highly dependent on the movement being studied as well as the locations in which markers are affixed to the skin (Leardini et al., 2005). We aim to quantify the effect of uncertainty in marker registration (McFadden et al., 2020) and model scaling (Kainz et al., 2017b) by generating ranges of equally plausible trajectories of joint angles, moments, and powers during human walking, thereby quantifying the effects of these sources of modeling uncertainty on the results of human gait analysis. We propose a methodology using only the motion capture data that are collected in typical human movement experiments and provide scripts in OpenSim so that others can easily reproduce our results and quantify the effect of modeling uncertainty in their own studies.

## 2 Methods

**FIGURE 1:**
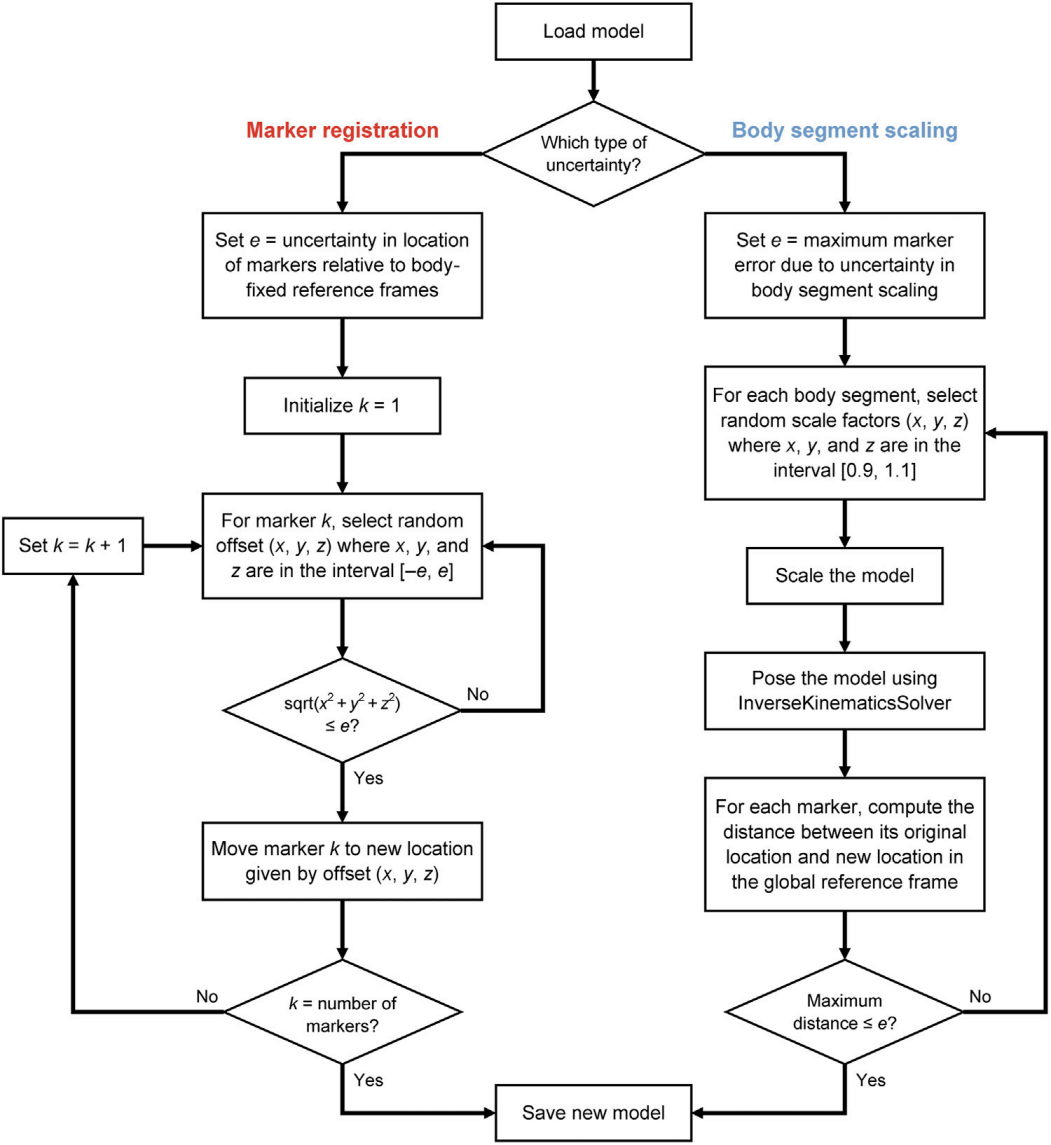
Uncertainty propagation process for generating a population of skeletal models that all yield marker positions within a specified level of uncertainty.

**FIGURE 2:**
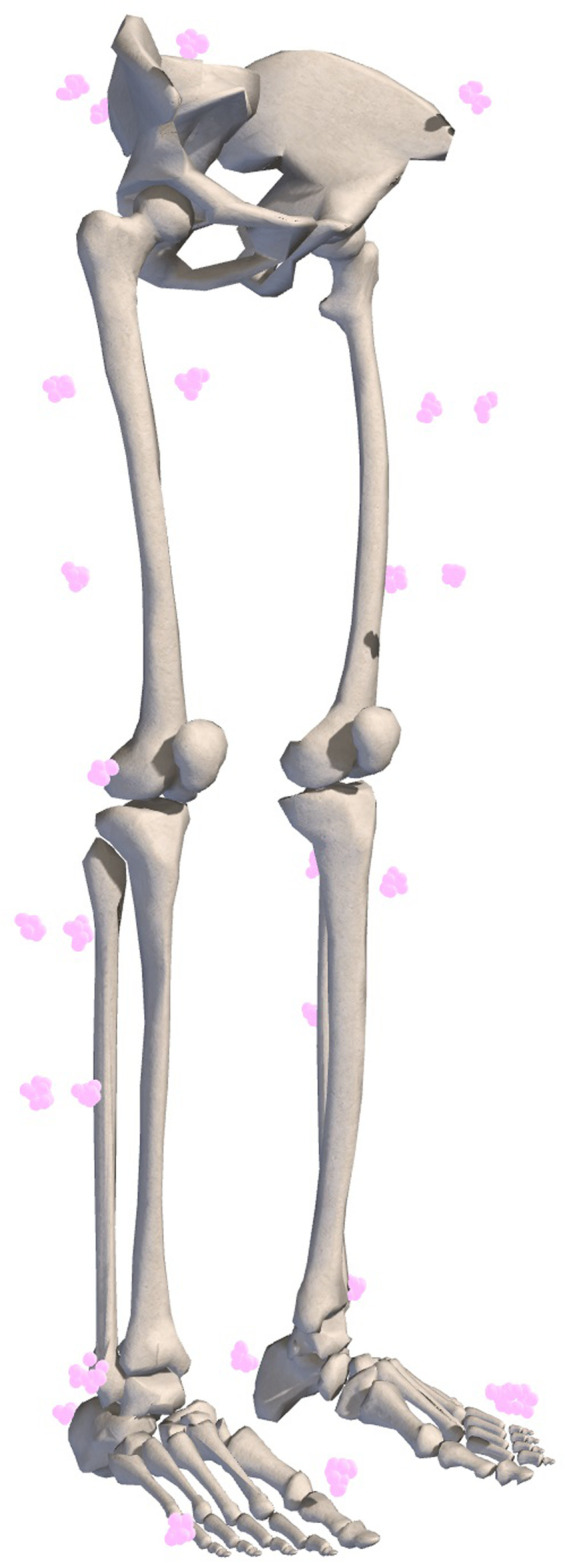
Example of marker registration uncertainty represented by equally probable marker locations. Ten marker sets with 1 cm uncertainty are shown.

To understand the effects of uncertainty on joint angles, moments, and powers during gait, we computed hundreds of IK solutions that satisfied a specified bound on marker uncertainty, *e* (Figure 1). For example, if *e* was set to 1 cm, then the model markers were no more than 1 cm away from their original locations after applying a random adjustment representing uncertainty in either marker registration or body segment scaling. We generated a set of *N* models to represent different marker registrations (Figure 1, left branch), but all within *e* of the original marker locations on the model, effectively defining sets of markers that fit within spheres of radius *e* centered on the original marker locations (Figure 2). With these models, we used OpenSim (Seth et al., 2018) to compute joint angles *via* IK and joint moments *via* inverse dynamics, and we estimated joint velocities to compute joint powers. Alternatively, model marker locations could differ from those on the original model due to model scaling (Figure 1, right branch). In this case, markers initially remained fixed relative to the anatomical reference frame on each body segment, but when the dimensions of that body segment were increased or decreased (“scaled”), the marker position with respect to the anatomical reference frame changed, which again resulted in models whose markers differed from those on the original model within a distance of *e*. Figure 1 summarizes the two strategies we used to generate new models; these strategies are described in detail below.

### 2.1 Generating Models With Marker Registration Uncertainty

Placement of mocap markers on an experimental subject varies with the individual being measured as well as the experimenter placing the markers. To capture this uncertainty in marker registration, we generated numerous models representing equally likely placements of markers on the OpenSim model. For a given gait model, we can generate an infinite set of unique models where each model contains an equally plausible set of registered markers that differ from the marker locations on the original model by a maximum distance (defined by our uncertainty parameter, *e*). For each marker in the model, its location was adjusted by adding a random perturbation in each spatial dimension from the interval $\left[-e,e\right]$ ; we then verified that the marker was within a distance of *e* of its original location. If the distance was greater, a new perturbation was selected. This selection strategy ensured that each point in a sphere of radius *e* was selected with equal probability, representing the uniform distribution of the marker registration uncertainty. The process was repeated until *N* models had been generated. We started at the lowest level of uncertainty (e.g., 0.5 cm), and with each subsequent increase in uncertainty, we augmented the previous set with models generated using the next value of *e* (e.g., 1.0 cm).

### 2.2 Generating Models With Body Segment Scaling Uncertainty

For a given gait model, we can generate an infinite set of unique models where each contains an equally plausible set of scaled body segments that only differ from those of the original model by their scale factors, and which result in model marker locations that differ from the original model marker locations by less than our uncertainty parameter *e*. For each body segment in the model, scale factors were selected at random from the interval $\left[90\%,110\%\right]$ ; the model was then posed using the InverseKinematicsSolver in OpenSim (Al Borno et al., 2021) to verify that each marker on the scaled model was within a distance of *e* of the corresponding marker on the original model. If any of the distances were greater, the set of body segment scale factors was discarded and new scale factors were randomly selected. The process was repeated until *N* models had been generated.

### 2.3 Analyses of OpenSim Models

We investigated the effect of uncertainty on the calculation of joint kinematics, kinetics, and powers during walking in a healthy individual (Dembia et al., 2017) and on the calculation of joint kinematics in patients with cerebral palsy walking in a crouch gait (Steele et al., 2013). For a typical subject from the first study (mass 83.5 kg, subject 14 walking at natural speed without carrying a load, trial 5, approximately one gait cycle), our “uncertainty propagation” process (Figure 1) was used to generate a variety of trajectories that reflect the underlying uncertainty due to marker registration and body segment scaling. Each model generated by the “uncertainty propagator” was run through the same processing pipeline as in the original study. In particular, for each walking model, we performed IK using the same marker weights and input marker data, resulting in a unique set of joint angle trajectories for each model.

In all cases, IK was used to compute joint angles and to evaluate the quality of each model and its joint angle trajectories based on its marker errors, which were computed with respect to the marker locations of the original model. Specifically, the error associated with each marker was determined at each instant in time during IK by computing the distance between its location (relative to ground) on the uncertainty-generated model and its location on the original model. For each uncertainty-generated model, the root-mean-square error (RMSE) was computed across all lower-body (pelvis and distal) markers at each instant in time and then averaged over time. The maximum value for this metric across all uncertainty-generated models was used to verify that the resulting IK solutions remained within the uncertainty margin of the original solution—that is, that the uncertainty-generated models represented equally plausible solutions. Similarly, the maximum marker error over all markers, instants in time, and models was computed and compared to the maximum marker error reported in the original study.

Each model and corresponding set of joint angle trajectories was used to compute inverse dynamics in OpenSim with the same set of measured ground reaction forces applied to the model. Joint angle trajectories were low-pass filtered at 6 Hz and differentiated to estimate joint velocities and accelerations within the Inverse Dynamics Tool. Resultant joint moments were then multiplied by the joint velocities to obtain the instantaneous joint powers for each model.

To investigate the effects of uncertainty on outcome measures of healthy walking (Dembia et al., 2017), we generated *N* = 100 models at each of four marker registration uncertainty levels and *N* = 100 models at each of four model scaling uncertainty levels; in each case, we used uncertainty levels (*e*) of 0.5, 1, 1.5, and 2 cm. In total, 800 models were generated for the study of healthy walking. For each model, nine peak values were extracted: three joint angles (peak hip extension angle, minimum knee flexion angle during stance, and peak ankle plantarflexion angle), three joint moments (peak hip flexion moment, peak knee flexion moment during stance, and peak ankle plantarflexion moment), and three joint powers (peak hip power at push off, minimum knee power during stance, and peak ankle power at push off). Because all models generated with a given uncertainty level were equally plausible, we simply collected the ranges of these peak values across all models at each level of uncertainty.

To investigate the effects of marker registration uncertainty on the classification of patients walking in a crouch gait (Steele et al., 2013), we generated *N* = 100 models at uncertainty levels of 1 and 2 cm for each of nine subjects: three originally classified as walking with a mild crouch (patients MI01, MI02, and MI03 in the original study), three with a moderate crouch (MO02, MO03, and MO04), and three with a severe crouch (SE01, SE02, and SE05). We then performed IK with each model and extracted the minimum knee flexion angle during stance for each leg. In total, 1800 models were generated for the study of crouch gait.

## 3 Results

Introducing uncertainty in model calibration due to marker registration (Table 1) and body segment scaling (Table 2) had predictable effects on marker errors, with the maximum RMSE across all models remaining below the uncertainty level. The maximum marker errors across all instants in time and all models were also within the maximum errors of the original models and their IK solutions. The results confirmed that the “uncertainty propagator” produced a set of models and joint angle trajectories that resulted in similar marker errors as were obtained in the original study, and did not artificially add to or inflate marker errors.

**TABLE 1:** The effect of marker registration uncertainty on marker errors (with respect to the marker locations on the original model) from IK for varying levels of uncertainty. For reference, the root-mean-square error (RMSE) for the original model with respect to experimental data was 2.1 cm and the maximum error across all markers and frames was 6.9 cm (Dembia et al., 2017).

Marker Registration Uncertainty (cm)	RMSE Averaged over all Frames, Maximum over all Models (cm)	Maximum Marker Error over all Frames and Models (cm)
0.5	0.44	1.32
1.0	0.87	2.42
1.5	1.28	3.13
2.0	1.74	4.38

**TABLE 2:** The effect of body segment scaling uncertainty on marker errors (with respect to the locations of the markers on the original model) from IK for varying levels of uncertainty.

Body Segment Scaling Uncertainty (cm)	RMSE Averaged over all Frames, Maximum over all Models (cm)	Maximum Marker Error over all Frames and Models (cm)
0.5	0.37	0.92
1.0	0.67	1.62
1.5	1.11	2.31
2.0	1.54	3.30

**FIGURE 3:**
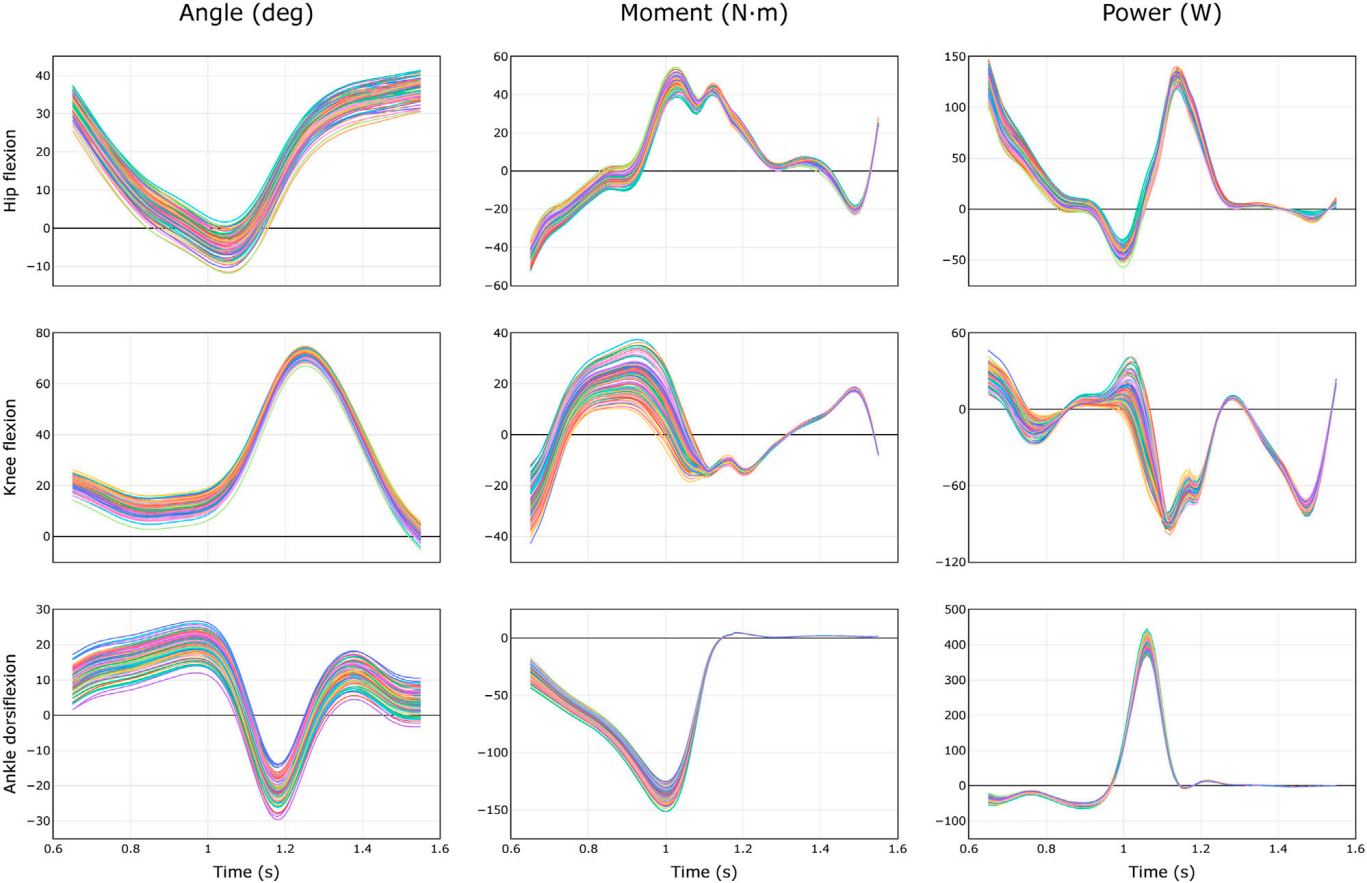
Equally probable trajectories of joint angles, moments, and powers during healthy walking (Dembia et al., 2017) due to uncertainty of 2 cm in marker registration. Results from *N* = 100 models are shown.

**FIGURE 4:**
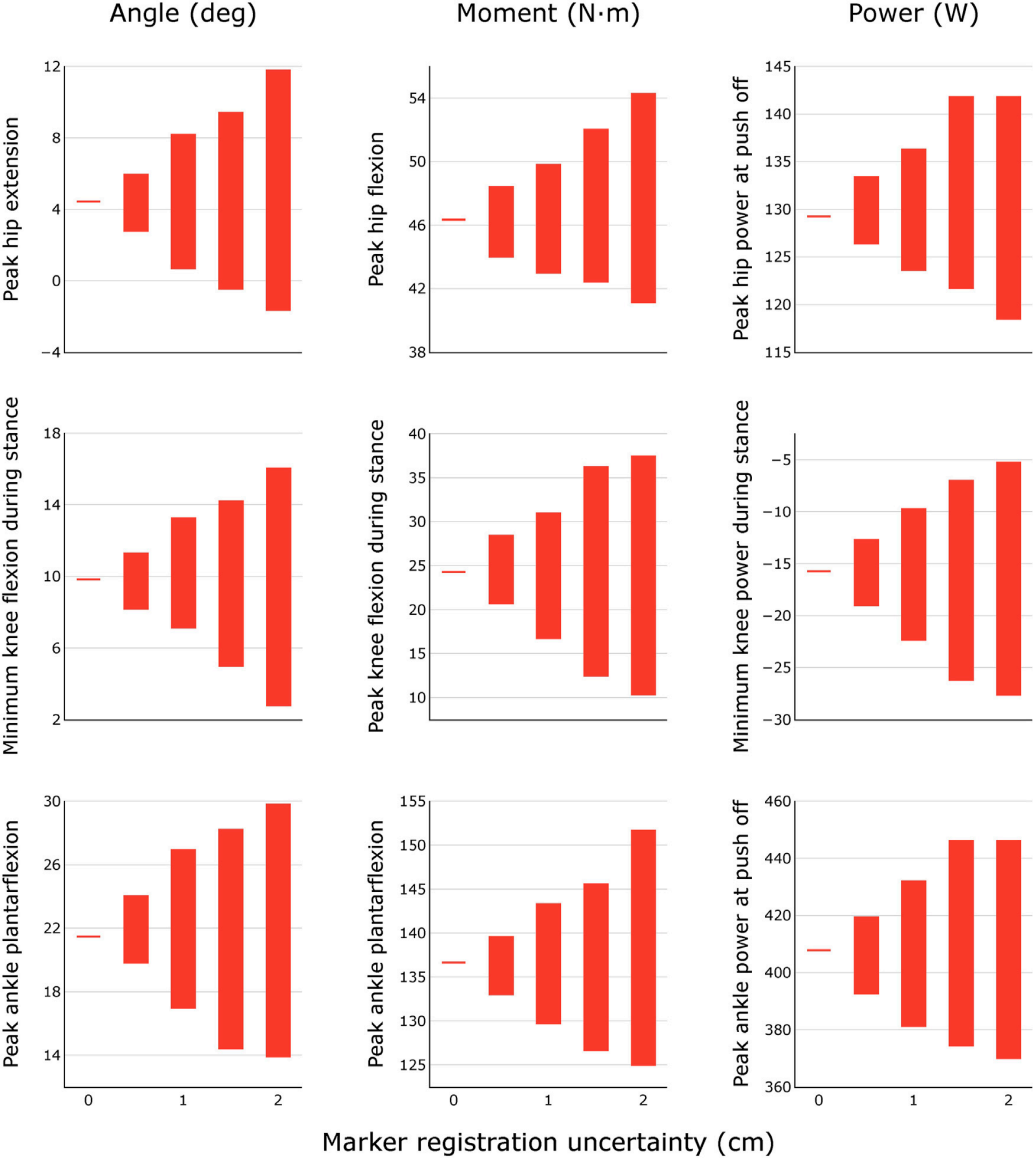
Variability in peak joint angles, moments, and powers during healthy walking for different levels of marker registration uncertainty. Results were obtained by generating *N* = 100 equally probable models at each level of uncertainty.

**FIGURE 5:**
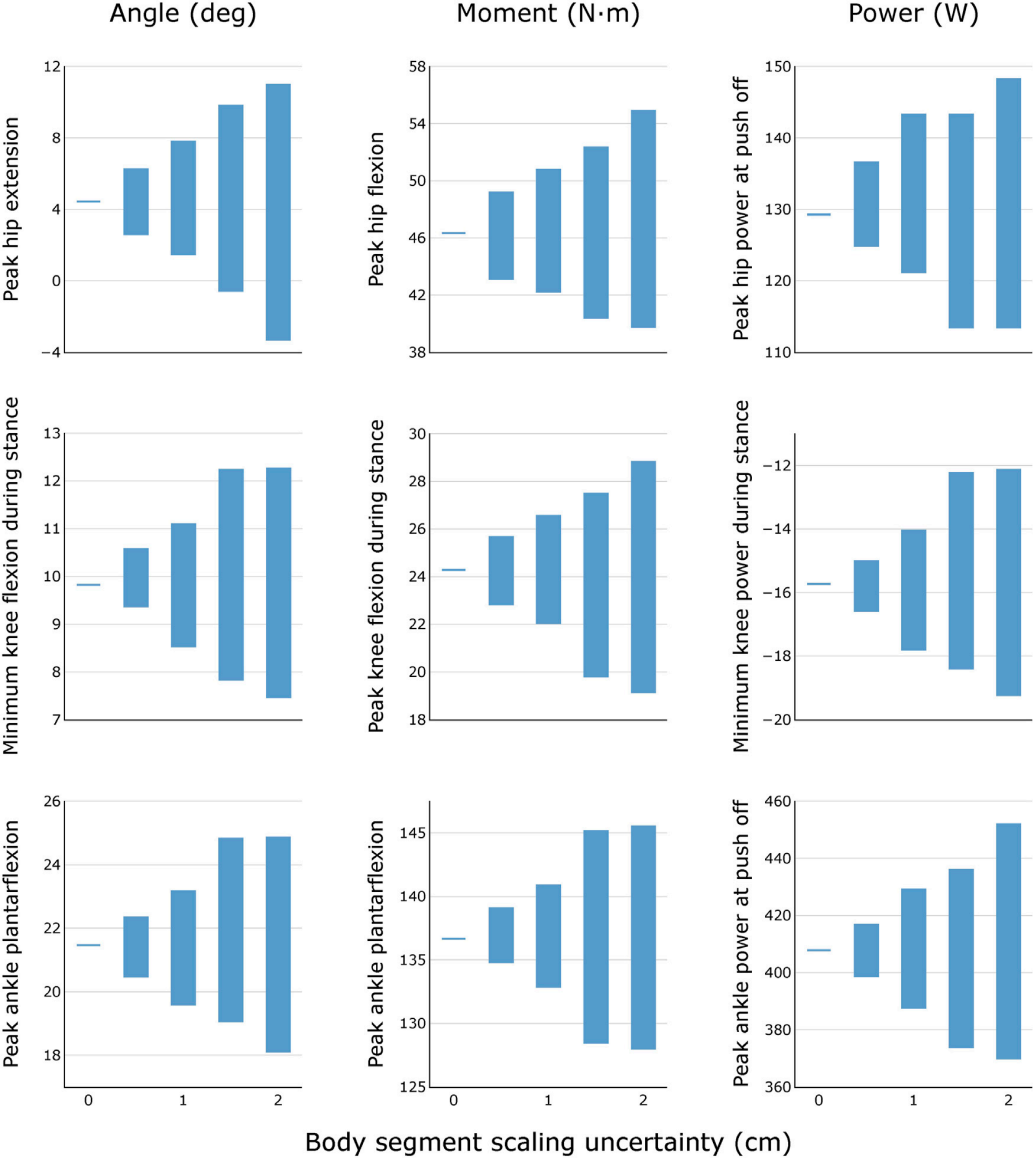
Variability in peak joint angles, moments, and powers during healthy walking for different levels of body segment scaling uncertainty. Results were obtained by generating *N* = 100 equally probable models at each level of uncertainty.

For each uncertainty level, the trajectories of model-estimated joint angles, moments, and powers spanned a substantial range (Figure 3). The range of peak angles, moments, and powers are shown for the hip, knee, and ankle over several levels of uncertainty in marker registration (Figure 4) and body segment scaling (Figure 5). The uncertainty in the peak ankle plantarflexion angle, peak ankle plantarflexion moment, and peak ankle power at push off due to marker registration uncertainty are provided in Table 3. For example, considering only marker registration uncertainty and at a level of only 2 cm, peak ankle plantarflexion angle varied by 15.9°, peak ankle plantarflexion moment varied by 26.6 N⋅m, and peak ankle power at push off varied by 75.9 W.

**TABLE 3:** Uncertainty in peak ankle angle, moment, and power during healthy walking for different levels of marker registration uncertainty. Results were obtained by generating *N* = 100 equally probable models at each level of uncertainty.

Marker Registration Uncertainty (cm)	Peak Ankle Plantarflexion Angle (deg)	Peak Ankle Plantarflexion Moment (N·m)	Peak Ankle Power at Push Off (W)
0.5	19.8–24.0	133.0–139.5	392.8–419.3
1.0	17.0–26.9	129.7–143.3	381.3–431.9
1.5	14.4–28.2	126.7–145.5	374.5–446.0
2.0	13.9–29.8	125.0–151.6	370.1–446.0

**FIGURE 6:**
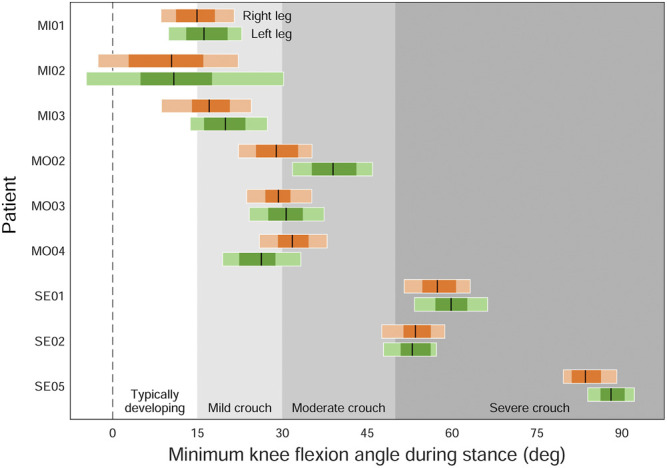
The effect of marker registration uncertainty on the classification of patients with cerebral palsy walking in a crouch gait (Steele et al., 2013). Individual legs were classified based on their minimum knee flexion angle (peak knee extension angle) during stance: typically developing (below 15°), mild crouch (15–30°), moderate crouch (30–50°), and severe crouch (above 50°). Black lines are the original classifications; dark and light horizontal bars indicate the ranges of minimum knee flexion angles resulting from 1 and 2 cm of uncertainty, respectively.

Propagating marker registration uncertainty onto the gait kinematics of children walking in a crouch gait (Figure 6) reveals that, for several classifications of crouch gait severity, we see equally likely solutions that cross the defined boundaries. For example, several “mild crouch” limbs could be classified equally well in the “typically developing” or “moderate crouch” categories.

## 4 Discussion

We quantified the effect of uncertainty in marker registration and model scaling by generating ranges of equally plausible trajectories of joint angles, moments, and powers during human walking. The marker registration uncertainty we explored can be considered a combination of uncertainty in the placement of physical markers on the subject as well as uncertainty in the placement of the corresponding markers on the OpenSim model. We demonstrated that peak joint angles could vary by up to 15.9° during healthy walking. When used to classify patients, this amount of uncertainty in joint kinematics could lead to misclassification of many individuals in a given study on crouch gait (e.g., 7 of 9 patients in Figure 6). The uncertainty in ankle push-off power (75.9 W or 0.91 W/kg) due to marker registration uncertainty exceeds the maximum range in estimated ankle plantarflexor powers between healthy and disabled elderly individuals (0.37 W/kg) (McGibbon and Krebs, 2004). Similarly, exoskeleton designers are concerned with increasing push-off power or reducing metabolic cost of transport by only a few percentage points (Collins et al., 2015; Uchida et al., 2016; Lee et al., 2017; Shepertycky et al., 2021), again within a range in which the uncertainty observed in this study would be significant.

One might wonder, “What about soft-tissue artifacts? Will STA not be a large contributor to uncertainty during model scaling and IK calculations?” Although STA is known to be an inescapable and substantial source of error in marker-based mocap (Stagni et al., 2005; Akbarshahi et al., 2010; Fiorentino et al., 2017, 2020; Lahkar et al., 2021), other sources of error that have received relatively less attention in the literature may be equally important. In this study, we have isolated and quantified the uncertainty due to errors in marker registration and model scaling. Surprisingly, even without explicit consideration of STA and even with only moderate amounts of marker error, the ranges in key outcome measures were larger than differences that are reported in many comparative studies. Our results have a profound effect on how we should interpret human movement results derived from marker-based motion capture measurements and inverse analyses. Although many studies do not report marker errors or the uncertainties associated with marker registration and model scaling, these sources of uncertainty do exist. Furthermore, soft-tissue artifacts, sensor noise, and other sources of error will only exacerbate the uncertainty in the outcome measures and, thus, will further increase the range of equally plausible results.

As we have demonstrated, uncertainty can easily result in incorrect classification of crouch gait severity or reporting success for an exoskeleton that provides only a few Watts of assistive push-off power. Although the details of the analyses we have performed may not apply to every study, we emphasize the importance of propagating errors throughout an inverse analysis to quantify the resulting uncertainty in the outputs of interest. Reporting incorrect conclusions and claims should be avoided as the consequent treatment decisions, investments in device design, or pursuits of clinical studies could have far-reaching effects on patient outcomes and research trajectories. Consequently, *we make the following recommendations* for movement scientists combining experimental marker data with models to estimate joint angles, moments, and/or powers in their research:

1) Perform studies with large numbers of participants to average out the uncertainty inherent in the results for each individual. Note that processing many gait trials from a single subject using a single calibrated model (with the same marker registration) will not address the issue, despite the impression that uncertainty has been adequately considered when a mean and standard deviation are computed or plotted over all trials.
2) Use the uncertainty propagation scripts we have provided to test the robustness of your study conclusions to a range of equally likely outcomes. Examine the uncertainty in your study conclusions relative to the uncertainties in the input data. Note that, unless subject-specific bone meshes are being used specifically to guide the placement of markers on the model, markers placed on models that are visualized using generic bone meshes may in fact appear to be misplaced.
3) For studies that involve a relatively small number of participants, generate a collection of equally plausible models for each subject, starting from a generic model and repeating the entire calibration process several times. For example, the model markers could be manually re-registered by several researchers.
4) When interpreting the results of inverse analyses, use categories and boundaries that separate the results into clinically meaningful groups while taking into account the effects of modeling uncertainty, rather than categorizing patients based on a single outcome measure. For example, Figure 6 indicates ambiguous classifications for several patient limbs when modeling uncertainty is considered.

There are three key limitations of this study. First, although marker registration and model scaling are not just issues when performing constrained inverse kinematics, we only tested kinematically constrained models—that is, we did not explicitly evaluate the effects of uncertainty using “unconstrained” modeling strategies (Solav et al., 2017). We also did not explore computing kinematics with data from inertial measurement units (IMUs), but we expect that similar effects would be observed due to the uncertainty in the placement of IMUs on the body. Second, we did not explore the effects of automated marker registration approaches, the residual reduction algorithm in OpenSim, or other error mitigation strategies such as compensation for soft-tissue motion (Dumas et al., 2015). Finally, the types and ranges of modeling uncertainties we examined are neither exhaustive nor necessarily applicable to every study. For example, the amount of registration uncertainty may differ among markers. However, the examples that we present demonstrate strategies that one could use to perform similar analyses in their studies. For studies requiring quantification of uncertainty over a large number of model parameters, Monte Carlo methods (e.g., (Myers et al., 2015)) may be used. In the future, we plan to assess the effects of uncertainty for unconstrained models, using different types of experimental data, and over a broader range of activities including movement of the upper extremity.

## Data Availability

We performed this study using the OpenSim software package, which is open source and freely available at https://simtk.org/projects/opensim. The data underlying the findings of this study are freely available at https://simtk.org/projects/quant_uncertain.
